# Cerebral microcirculation is impaired during sepsis: an experimental study

**DOI:** 10.1186/cc9205

**Published:** 2010-07-28

**Authors:** Fabio Silvio Taccone, Fuhong Su, Charalampos Pierrakos, Xinrong He, Syril James, Olivier Dewitte, Jean-Louis Vincent, Daniel De Backer

**Affiliations:** 1 Department of Intensive Care, Erasme Hospital, Université Libre de Bruxelles, Route de Lennik, 808, 1070 - Bruxelles, Belgium; 2 Department of Neurosurgery, Erasme Hospital, Université Libre de Bruxelles, Route de Lennik, 808, 1070 - Bruxelles, Belgium

## Abstract

**Introduction:**

Pathophysiology of brain dysfunction due to sepsis remains poorly understood. Cerebral microcirculatory alterations may play a role; however, experimental data are scarce. This study sought to investigate whether the cerebral microcirculation is altered in a clinically relevant animal model of septic shock.

**Methods:**

Fifteen anesthetized, invasively monitored, and mechanically ventilated female sheep were allocated to a sham procedure (*n *= 5) or sepsis (*n *= 10), in which peritonitis was induced by intra-abdominal injection of autologous faeces. Animals were observed until spontaneous death or for a maximum of 20 hours. In addition to global hemodynamic assessment, the microcirculation of the cerebral cortex was evaluated using Sidestream Dark-Field (SDF) videomicroscopy at baseline, 6 hours, 12 hours and at shock onset. At least five images of 20 seconds each from separate areas were recorded at each time point and stored under a random number to be analyzed, using a semi-quantitative method, by an investigator blinded to time and condition.

**Results:**

All septic animals developed a hyperdynamic state associated with organ dysfunction and, ultimately, septic shock. In the septic animals, there was a progressive decrease in cerebral total perfused vessel density (from 5.9 ± 0.9 at baseline to 4.8 ± 0.7 n/mm at shock onset, *P *= 0.009), functional capillary density (from 2.8 ± 0.4 to 2.1 ± 0.7 n/mm, *P *= 0.049), the proportion of small perfused vessels (from 95 ± 3 to 85 ± 8%, *P *= 0.02), and the total number of perfused capillaries (from 22.7 ± 2.7 to 17.5 ± 5.2 n/mm, *P *= 0.04). There were no significant changes in microcirculatory flow index over time. In sham animals, the cerebral microcirculation was unaltered during the study period.

**Conclusions:**

In this model of peritonitis, the cerebral microcirculation was impaired during sepsis, with a significant reduction in perfused small vessels at the onset of septic shock. These alterations may play a role in the pathogenesis of septic encephalopathy.

## Introduction

Sepsis and septic shock still represent major health issues, with persisting high morbidity and mortality rates in critically ill patients [1]. Sepsis is associated with tissue hypoperfusion and metabolic impairment, which may contribute to the associated multiple organ failure [2]. Cerebral dysfunction occurs commonly during severe sepsis, but its pathophysiology remains poorly understood [3]. Inflammation, blood-brain barrier (BBB) abnormalities, impairment of astrocytes and neurons, neurotransmitter derangements and apoptosis may all be involved [4]; nevertheless, some autopsy reports in patients who died in refractory septic shock described diffuse cerebral ischemic lesions, suggesting that impaired oxygen delivery to the brain could be involved in the development of sepsis-associated encephalopathy (SAE) [5]. As the brain is very dependent on an appropriate blood supply, some studies have suggested that reduced cerebral blood flow (CBF) [6] or disturbed cerebral autoregulation [7,8] may be implicated in the pathogenesis of SAE. However, brain dysfunction during sepsis may occur even when global hemodynamics seem to be adequate [9], and microcirculatory failure may, therefore, play a role in the occurrence of SAE [4].

Microcirculatory perfusion is responsible for the fine-tuning of the oxygen supply to the organs [10] and microcirculatory alterations may play a key role in the pathogenesis of sepsis-related organ dysfunction [11,12]. Sepsis-associated microcirculatory alterations include a decrease in capillary density and an increased heterogeneity of blood flow with perfused capillaries in close proximity to stopped or intermittently-perfused capillaries [10]. These alterations have been reported in the sublingual area [13-16], but similar findings have also been described in experimental models of sepsis in many organs, including striated muscle, small bowel mucosa and liver [17-20].

The impact of sepsis on the brain microcirculation is not well defined. Some animal studies described alterations in the cerebral microvascular network during sepsis [21-25] but these studies were limited by several factors. First, the laser Doppler techniques used to assess the microcirculation are unable to discriminate capillary flow from flow in other microvessels [21,22]. Second, these studies observed animals for a short period of time thus limiting extrapolation of these results to the entire time course of the septic process. Third, the model used was not always clinically relevant because of the limited amount of fluid resuscitation and the absence of a hyperkinetic phase.

We evaluated the occurrence of microcirculatory alterations during sepsis in a clinically relevant ovine model of sepsis induced by fecal peritonitis. We used the sidestream dark field (SDF) imaging technique, a modified orthogonal polarization spectral (OPS) technology [26], which has been successfully used to study the cerebral microcirculation in experimental models of cardiogenic and hemorrhagic shock [27,28] and cardiac arrest [29]. We hypothesized that the cerebral microcirculation may be impaired during sepsis and that these alterations would be unrelated to the global hemodynamic changes.

## Materials and methods

The study protocol was approved by the Institutional Review Board for Animal Care of the Free University of Brussels, Brussels, Belgium. Care and handling of the animals followed National Institutes of Health guidelines [30].

### Experimental animals

Twelve female sheep, weighing between 27 and 35 kg, were fasted for 24 hours with free access to water prior to the experiment. On the day of the experiment, the animals were premedicated with intramuscular midazolam (0.25 mg/kg, Dormicum, Roche SA, Beerse, Belgium) and ketamine hydrochloride (20 mg/kg, Imalgine, Merial, Lyon, France) and then placed in the supine position. The cephalic vein was cannulated with a peripheral venous 18-gauge catheter (Surflo IV Catheter, Terumo Medical Company, Leuven, Belgium). An intravenous administration of 30 μg/kg fentanyl citrate (Janssen, Beerse, Belgium) and 0.1 mg/kg rocuronium bromide (Esmeron, Organon, Oss, The Netherlands) was used for endotracheal intubation (8 mm endotracheal tube, Hi-Contour, Mallinckrodt Medical, Athlone, Ireland). All sheep were sedated with a continuous intravenous administration of midazolam (0.2 mg·kg^-1^·hr^-1^), ketamine hydrochloride (0.5 mg·kg^-1^·hr^-1^) and morphine (0.2 mg·kg^-1^·hr^-1^). Muscular blockade was achieved using 10 μg·kg^-1^·hr^-1 ^of rocuronium throughout the experiment to avoid movement artefacts. Boluses of fentanyl (5 mg) were administered if needed in case of tachycardia and/or hypertension suggesting insufficient anesthesia. Mechanical ventilation (Servo 900 C ventilator; Siemens-Elema, Solna, Sweden) was begun with the following settings: tidal volume of 10 mL/kg, respiratory rate of 12 to 16 breaths/minute, positive end-expiratory pressure of 5 cm H_2_O, an inspired oxygen fraction (FiO_2_) of 1, inspiratory time to expiratory time ratio of 1:2 and a square-wave pattern. Respiratory rate was adjusted to maintain end-tidal carbon dioxide pressure (PetCO_2_, 47210 A Capnometer; Hewlett Packard GmbH, Boehlingen, Germany) between 35 and 45 mmHg before arterial cannulation was established. A 60 cm plastic tube (inner-diameter 1.8 cm) was inserted into the stomach to drain its content and to prevent rumen distension. A 14F Foley catheter (Beiersdorf AG, Hamburg, Germany) was placed to record the urinary output throughout the experiment.

### Surgical procedures

The right femoral artery and vein were surgically exposed. A 6F arterial catheter (Vygon, Cirencester, UK) was invasively introduced into the femoral artery and connected to a pressure transducer (Edwards Lifescience, Irvine, CA, USA) zeroed at mid-chest level. An introducer was inserted through the femoral vein, and a 7F Swan-Ganz catheter (Edwards Lifesciences) was advanced into the pulmonary artery. A midline laparotomy was then performed in 10 animals (sepsis group). After cecotomy, 1.5 g/kg body weight of feces was collected. The cecum was then closed and the area around the cut disinfected with iodine solution. An additional suture was performed to prevent contamination and the cecum was returned to the abdominal cavity. A large plastic tube was inserted through the abdominal wall for later injection of feces. The abdomen was then closed in two layers. During the surgical operation, Ringer's lactate and 6% hydroxyethyl starch (HES) solutions were infused at rates of 1 mL·kg^-1^·hr^-1 ^and 2 mL·kg^-1^·hr^-1^, respectively. After abdominal surgery, the animals were turned to the prone position and allowed to stabilize before baseline measurements were recorded. Bilateral craniotomy was performed in all animals using a high-speed drill and a fine wire saw (Aesculap-Werke AG, Tuttlingen, Germany) to open two holes and connect them until a segment of bone (bone flap) of about 3 × 3 cm was created in the sheep's frontal bone. The dura covering the frontal lobes was then opened in a large incision carefully avoiding any cortical damage. The left and right frontal lobes were exposed and bleeding from the skull was controlled using surgical wax. At the end of the procedure, the skin flaps were sutured in place. The craniotomy holes were then protected by wet sterile gauzes, avoiding any contact with the brain cortex. Brain desiccation was prevented by local hourly administration of 2 mL saline solution. Five animals, in which all experimental procedures were performed except laparotomy and feces injection, served as sham controls.

### Monitoring and measurements

Volume-controlled mechanical ventilation was adjusted to ensure normoxia (80 mmHg ≤PaO_2 _≤120 mmHg) and normocapnia (35 mmHg ≤PaCO_2 _≤45 mmHg) according to repeated blood gas analysis (ABL500; Radiometer, Copenhagen, Denmark). Hemoglobin concentration and oxygen saturation were measured with an analyzer calibrated for ruminant animals (OSM3; Radiometer). Peak airway pressure, plateau airway pressure, expiratory gas flow and FiO_2 _were recorded hourly. Thoracopulmonary compliance was calculated using a standard formula. Arterial samples were obtained at baseline and then hourly after feces spillage. The total amount of blood withdrawn for analyses was around 60 mL (that is, around three percent of each sheep's estimated total blood volume). All monitored variables were recorded every 60 minutes. Measurements of mean arterial pressure (MAP), pulmonary arterial pressure, right atrial pressure, and pulmonary artery occlusion pressure (PAOP) were obtained at end-expiration (Sirecust 404; Siemens, Erlangen, Germany). Core temperature and cardiac output (Vigilance; Baxter, Edwards Critical Care) were continuously monitored. Body surface area [31], cardiac index, stroke volume index, and systemic vascular resistance (SVR) were calculated using standard formulas.^.^

### Cerebral microcirculation

The microvascular network of the cerebral cortex was visualized using an SDF videomicroscopy system (MicroScan^TM^, MicroVisionMedical Inc, Amsterdam, Netherlands), with a 5× imaging objective giving 326× magnification. The lens of the imaging device was covered with a disposable sterile cap and was applied without pressure to the cerebral frontal cortex. Because of brain pulsatility, this was best accomplished by placing the device on a metallic arm for stabilization (Giesseci, Avellino, Italy). The absence of pressure was ensured by preservation of flow in large vessels [32]. Guided by previous published studies in the same model [33], images were recorded at times 0 (baseline), 6 hours (corresponding to the hyperdynamic phase) and 12 hours (corresponding to the onset of organ dysfunctions). In view of the fact that some animals may die earlier than 18 hours after feces injection and that global hemodynamics in the late phase of sepsis vary from one animal to the other [33], the last time point was considered at shock onset (defined as MAP <65 mmHg refractory to fluid administration and lactate level >2.0 mmol/L). At least five videostrips from different areas, each of minimum duration of 20 seconds, were recorded on disk, using a computer and a video card (MicroVideo; Pinnacle Systems, Mountain View, CA, USA). The images were then stored under a random number for further analysis. An investigator blinded to group allocation and time later analyzed these sequences semi-quantitatively [13,32]. In brief, three equidistant horizontal and three equidistant vertical lines were drawn. The vascular density was calculated as the number of vessels crossing these lines divided by the total length of the lines. The type of flow was defined as continuous, intermittent or absent [32]. To compute the Mean Flow Index (MFI), vessels with continuous flow were further divided into normal and sluggish [34]. Vessel size was determined using a micrometer scale and the vessels were separated into large and small vessels, using a diameter cutoff value of 20 μm [32]. Small vessel perfusion was defined as the proportion of small perfused vessels (PSPV), and calculated as the number of capillaries continuously perfused during the 20-second observation period divided by the total number of vessels of the same type. Small perfused vascular density (FCD) was calculated as the product of capillary density and perfused vessel density of vessels of same type. The Heterogeneity Index for both MFI and PSPV [15] was also calculated. In each animal, the data from the investigated areas were averaged for each time point.

### Experimental protocol

After the surgical procedures, baseline measurements, including cerebral microcirculation, were obtained. Feces were then spilled into the abdominal cavity. In feces samples from earlier experiments in this model, the bacterial load was of similar magnitude in all animals (unpublished data). Ringer's lactate and 6% HES solutions were infused at a rate titrated to avoid hypovolemia and to maintain the PAOP at baseline level. All animals were observed until spontaneous death or for a maximum of 20 hours after the induction of peritonitis.

### Statistical analysis

Statistical analysis was performed using SPSS 13.0 for Windows (2004; SPSS Inc, Chicago, IL, USA). Data are presented as mean ± SD or median (range). Variables were compared with parametric Student's *t*-test or the Mann-Whitney U test for nonparametric data. Two-way ANOVA for groups per time interaction and group and time comparisons, with Bonferroni correction for *post hoc *analysis, was used. Linear correlation was calculated using Spearman correlation coefficient. A *P-*value of <0.05 was considered statistically significant.

## Results

**Figure 1:**
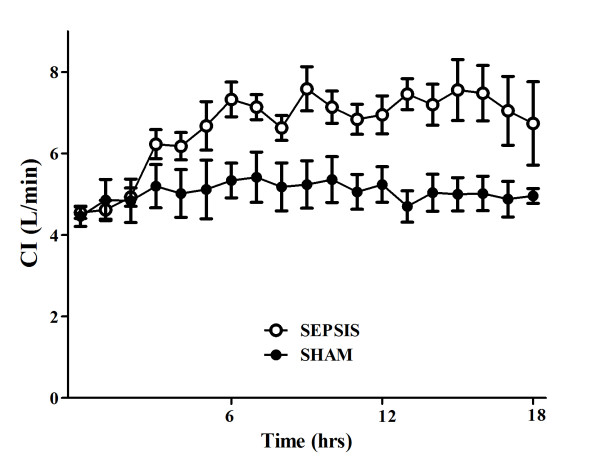
**Evolution over time of cardiac index (CI) in sham (*n *= 5) and septic (*n *= 10) animals**.

**Figure 2:**
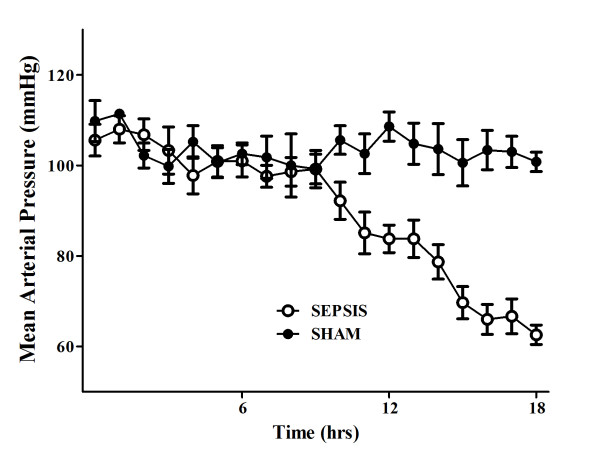
**Evolution over time of mean arterial pressure (MAP) in sham (*n *= 5) and septic (*n *= 10) animals**.

**Figure 3:**
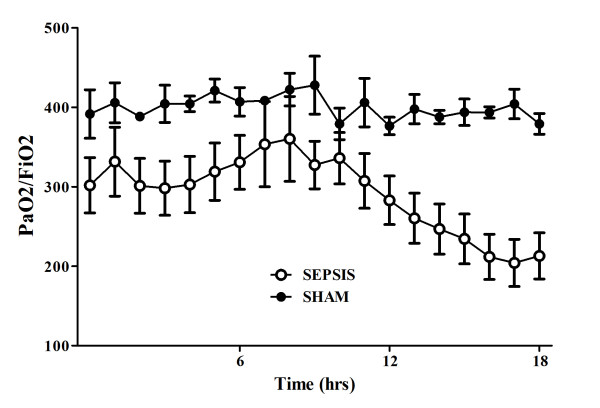
**Evolution over time of PaO2/FiO2 ratio in sham (*n *= 5) and septic (*n *= 10) animals**.

**Figure 4:**
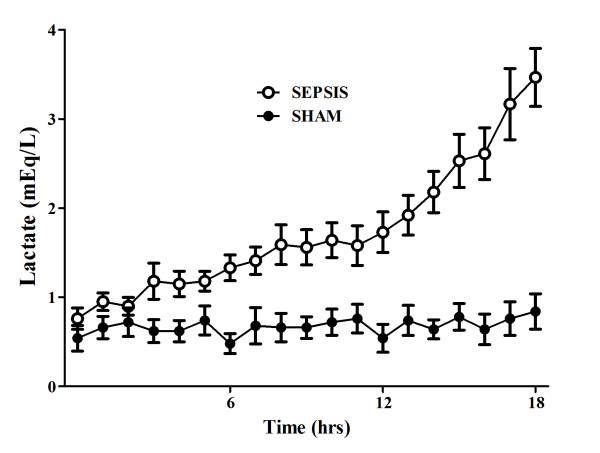
**Evolution over time of lactate levels in sham (*n *= 5) and septic (*n *= 10) animals**.

There were no differences in baseline global or regional hemodynamic values between the sham and septic animals (Tables 1 and 2). The septic animals developed three distinctive states over time. Three hours after sepsis induction, these animals developed a hyperdynamic state, characterized by an increased cardiac index and relatively preserved MAP without obvious signs of organ dysfunction (Figures 1, 2, 3 and 4). After 11 to 14 hours, the PaO_2_/FiO_2 _ratio and thoracopulmonary compliance decreased, indicating sepsis-related pulmonary dysfunction. Finally, lactic acidosis and refractory hypotension occurred in all animals (15 hours after feces injection in three, after 18 hours in seven animals). The hyperdynamic state persisted until the agonal phase. All animals died after 18 to 20 hours, except two which died after 16 hours. The five sham animals had stable MAP, CI, and pulmonary function throughout the study period (Figures 1, 2, 3 and 4).

**Table 1:** Evolution of systemic hemodynamics and acid-base variables over time in the septic (*n *= 10) and the sham (*n *= 5) animals

		Baseline	6 hours	12 hours	18 hours^#^	ANOVA	
						
						Time (*P*)	Groups (*P*)
**Temperature, °C**	Sepsis	39.5 ± 0.5	39.9 ± 0.8	40.2 ± 1.1^a,b^	41.2 ± 0.8^a,b,c ^*	0.0002	0.01
	Sham	39.6 ± 0.3	39.4 ± 0.5	39.7 ± 0.7	40.4 ± 0.5	0.65	

**HR, beats/min**	Sepsis	116 ± 27	137 ± 22^a^	138 ± 14^a^	140 ± 15^a^	0.05	0.38
	Sham	108 ± 15	132 ± 13	132 ± 14	133 ± 8^a^	0.04	

**Cardiac Index, L/min.m^2^**	Sepsis	4.5 ± 0.5	7.3 ± 1.3^a ^*	6.9 ± 1.5^a ^*	5.6 ± 1.3	< 0.0001	< 0.0001
	Sham	4.5 ± 0.6	5.3 ± 1.0	5.2 ± 1.0	5.0 ± 0.4	0.21	

**MAP, mmHg**	Sepsis	106 ± 11	101 ± 11	84 ± 10^a ^*	56 ± 6^a,b ^*	< 0.0001	0.0002
	Sham	112 ± 11	105 ± 9	107 ± 10	100 ± 4	0.32	

**MPAP, mmHg**	Sepsis	15 ± 4	14 ± 2	19 ± 4^a,b^	20 ± 4^a,b^	< 0.0001	0.06
	Sham	16 ± 1	15 ± 3	16 ± 3	17 ± 5	0.86	

**SVRI, dynes.sec.cm^-5^**	Sepsis	1853 ± 211	1120 ± 248^a^	960 ± 211^a ^*	777 ± 176^a,b ^*	< 0.0001	< 0.0001
	Sham	1907 ± 253	1540 ± 292	1597 ± 273	1691 ± 138	0.1	

**PF ratio**	Sepsis	302 ± 110	326 ± 113	283 ± 96 *	197 ± 91^b ^*	0.01	< 0.0001
	Sham	392 ± 68	407 ± 40	377 ± 24	379 ± 29	0.69	

**PaCO2, mmHg**	Sepsis	41 ± 3	39 ± 2	41 ± 4	46 ± 9^b^	0.009	0.11
	Sham	37 ± 3	39 ± 3	39 ± 4	42 ± 4	0.09	

**TPC, mL/mmHg**	Sepsis	18 ± 2	19 ± 4	15 ± 4	12 ± 3^a,b^	0.0002	0.23
	Sham	18 ± 3	18 ± 1	17 ± 2	15 ± 3	0.22	

**Urine output, mL/hr**	Sepsis	96 ± 36	105 ± 100	99 ± 85	127 ± 65	0.53	0.053
	Sham	79 ± 25	128 ± 45	155 ± 99	95 ± 35	0.48	

**Fluid amount, mL**	Sepsis	314 ± 110	1635 ± 596^a ^*	1705 ± 497^a ^*	2210 ± 854^a,b,c ^*	< 0.0001	< 0.0001
	Sham	218 ± 65	265 ± 93	310 ± 74	278 ± 61	0.81	

**Hemoglobin, g/dL**	Sepsis	10.7 ± 1.1	10.9 ± 1.6	11.1 ± 1.2	11.6 ± 1.6	0.56	0.66
	Sham	10.5 ± 0.7	10.5 ± 0.8	11.3 ± 0.7	11.4 ± 0.7	0.49	

**Lactate (mEq/L)**	Sepsis	0.9 ± 0.3	1.3 ± 0.5	1.7 ± 0.7^a ^*	4.0 ± 1.2^a,b,c ^*	< 0.0001	< 0.0001
	Sham	0.7 ± 0.2	0.6 ± 0.3	0.7 ± 0.2	0.8 ± 0.4	0.71	

Data are presented as mean ± SD.

^# ^Shock onset for septic group.

°C, Celsius degrees; HR, heart rate; CI, cardiac index; MAP, mean arterial pressure; MPAP, mean pulmonary arterial pressure; SVRI, systemic vascular resistance index; PF, PaO2/FiO2; TPC, thoraco-pulmonary compliance.

No interference between time and groups was found for all the studied variables in ANOVA analysis. Significant at 5% vs. baseline (^a^), vs 6 hours (^b^) or vs 12 hours (^c^) or significant at 5% vs. sham (*) in *post-hoc *Bonferroni correction.

**Table 2:** Evolution in cerebral microcirculation over time in the septic (*n *= 10) and the sham (*n *= 5) animals

		Baseline	6 hours	12 hours	18 hours^#^	ANOVA	
						
						Time (*P*)	Groups (*P*)
**TVD**	Sepsis	6.1 ± 0.8	5.4 ± 0.5	5.7 ± 0.8	5.4 ± 0.6	0.15	0.61
	Sham	5.8 ± 0.8	5.9 ± 0.7	5.6 ± 0.8	5.8 ± 0.6	0.65	

**TPVD**	Sepsis	5.9 ± 0.9	5.1 ± 0.5	5.1 ± 0.6	4.8 ± 0.7^a^	0.009	0.14
	Sham	5.7 ± 0.7	5.8 ± 0.7	5.5 ± 0.7	5.5 ± 0.5	0.76	

**NPC**	Sepsis	22.7 ± 2.8	18.8 ± 3.2 *	18.7 ± 5.9 *	17.5 ± 5.2 *^a^	0.048	< 0.001
	Sham	25.1 ± 0.9	24.7 ± 2.3	23.8 ± 3.3	25.8 ± 0.8	0.42	

**MFI**	Sepsis	2.9 ± 0.1	2.9 ± 0.1	2.9 ± 0.1	2.7 ± 0.3	0.21	0.87
	Sham	2.9 ± 0.1	2.9 ± 0.1	2.9 ± 0.1	2.8 ± 0.2	0.33	

**PSPV - HI**	Sepsis	0.11 ± 0.06 *	0.26 ± 0.15 *^a^	0.23 ± 0.11 *	0.32 ± 0.13 *^a^	0.002	< 0.001
	Sham	0.04 ± 0.01	0.03 ± 0.01	0.03 ± 0.02	0.06 ± 0.04	0.25	

**MFI - HI**	Sepsis	0.13 ± 0.07	0.18 ± 0.13	0.23 ± 0.14 *^a^	0.25 ± 0.14 *^a^	0.003	0.002
	Sham	0.07 ± 0.06	0.18 ± 0.09	0.08 ± 0.05	0.13 ± 0.07	0.19	

Data are presented as mean ± SD.

^# ^Shock onset for septic group.

Significant at 5% vs. baseline (^a^), vs 6 hours (^b^) or vs 12 hours (^c^). Significant at 5% vs. sham (*).

TVD, total vessel density; TPVD, total perfused vessel density; PSPV, proportion of small perfused vessels; FCD, functional capillary density; NPC, number of perfused capillaries; MFI, mean flow index; HI, heterogeneity index; (*P*) = *P-*value.

No interference between time and groups was found for all the studied variables in ANOVA analysis. Significant at 5% vs. baseline (^a^), or vs. sham (*) in *post-hoc *Bonferroni correction.

**Figure 5:**
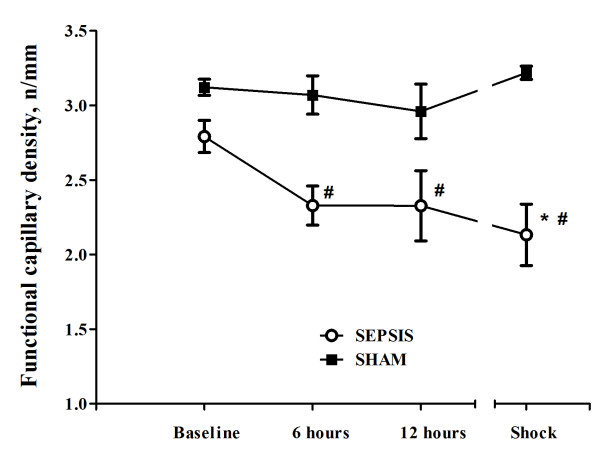
**Changes in cerebral functional capillary density (FCD) in the septic (*n *= 10) and the sham (*n *= 5) animals**. Data are presented as mean ± SD. ANOVA analysis for FCD: *P *= 0.049 (time, sepsis group) and *P *< 0.001 (group). ANOVA analysis for PSPV: *P *= 0.02 (time, sepsis group) and *P *< 0.001 (group). *P*-value <.05 vs. baseline (*) or vs. sham (^#^) in *post-hoc *Bonferroni correction.

**Figure 6:**
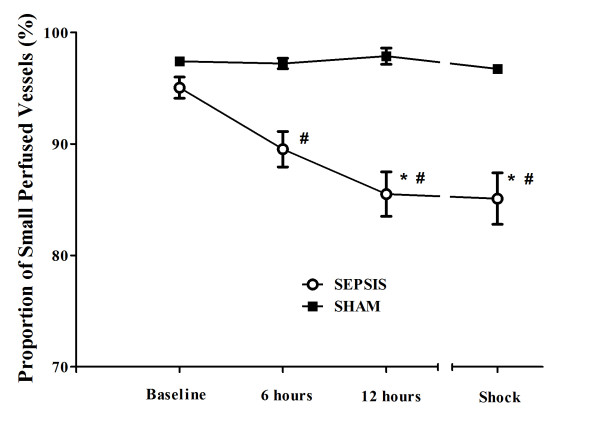
**Changes in proportion of small perfused vessels (PSPV) in the septic (*n *= 10) and the sham (*n *= 5) animals**. Data are presented as mean ± SD. ANOVA analysis for FCD: *P *= 0.049 (time, sepsis group) and *P *< 0.001 (group). ANOVA analysis for PSPV: *P *= 0.02 (time, sepsis group) and *P *< 0.001 (group). *P-*value <.05 vs. baseline (*) or vs. sham (^#^) in *post-hoc *Bonferroni correction.

**Figure 7:**
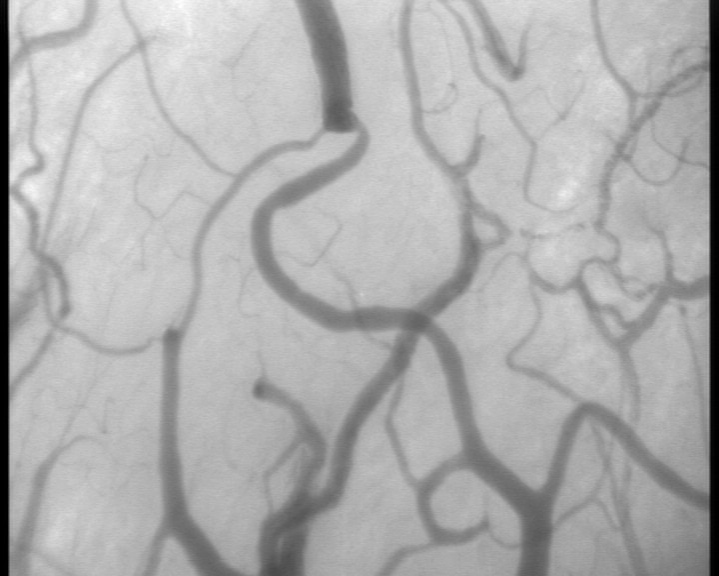
**Digital photomicrographs of the cerebral cortical microcirculation of a septic animal at baseline**.

**Figure 8:**
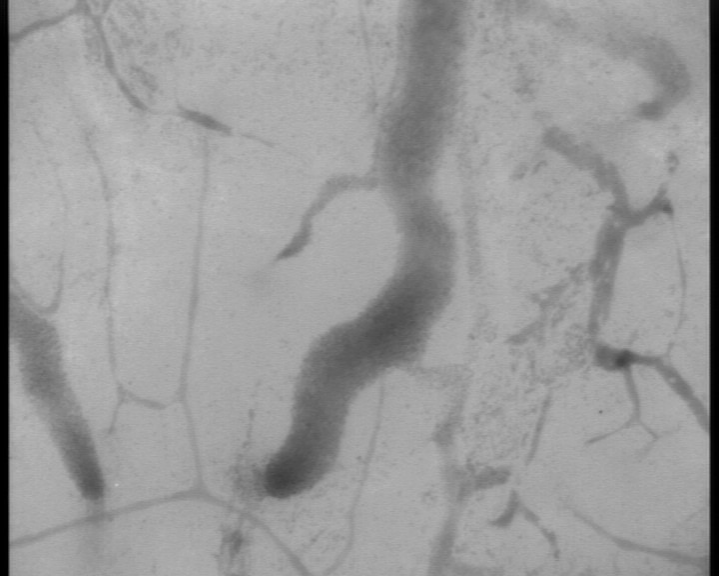
**Digital photomicrographs of the cerebral cortical microcirculation of a septic animal at shock onset**.

**Figure 9:**
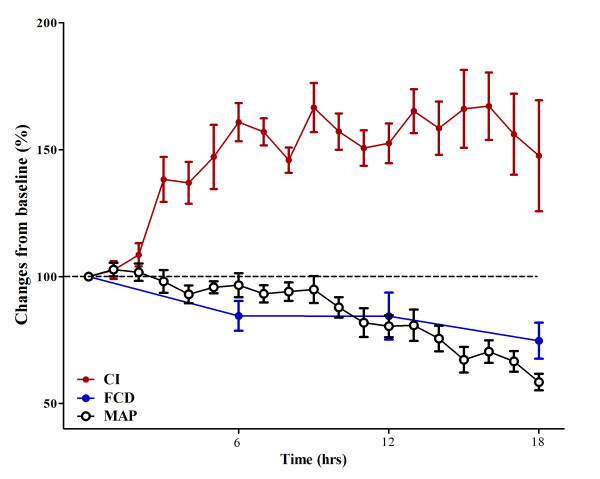
**Correlation between microcirculation and global hemodynamics**. Changes from baseline (100%) of cardiac index (CI; red circles), mean arterial pressure (MAP; white circles) and functional capillary density (FCD; blue circles) over the study period. Changes in FCD appear to occur earlier than significant changes in MAP and already during the hyperdynamic phase.

Changes in cerebral microcirculation at different time points are shown in Table 2 and Figures 5 and 6. Typical images of sheep cerebral microcirculation at baseline and at shock onset are shown in Figures 7 and 8. The development of septic shock was associated with decreases in cerebral total PVD (from 5.9 ± 0.9 to 4.8 ± 0.7 n/mm, *P *= 0.009), FCD (from 2.8 ± 0.4 to 2.1 ± 0.7 n/mm, *P *= 0.049), PSPV (from 95 ± 3 to 85 ± 8%, *P *= 0.02) (Figure 3) and the total number of perfused capillaries (from 22.7 ± 2.7 to 17.5 ± 5.2 n/mm, *P *= 0.04). PSPV was already decreased at 12 hours (to 85 ± 6%, *P *= 0.02 vs. baseline). There were no significant changes in total vessel density (TVD) or MFI during the experiment. The heterogeneity index of both MFI and PSPV increased over time (Table 2). The decrease in FCD was not correlated to the changes in CI, MAP or arterial lactate levels (Figure 9). In sham animals, the cerebral microcirculation was unaltered over the study period.

## Discussion

The key finding in this study is that the cortical cerebral microcirculation is altered in this hyperdynamic model of sepsis. These microcirculatory abnormalities became significant at the onset of septic shock, and were not prevented by aggressive fluid administration. Interestingly, the variability of FCD and PSPV in septic animals was greater than the variability in sham animals already at six hours after sepsis induction, suggesting that alterations in cerebral microcirculation occur earlier than shown only by the analysis of individual variations. Moreover, changes in the cerebral microcirculation were not related to changes in MAP, CI or lactate, suggesting that these alterations in the brain may occur even when global perfusion pressure is maintained, such as in non-hypotensive conditions.

These findings provide additional evidence of a dissociation between macro- and microcirculations, as reported for the sublingual area in human sepsis [13,32] and suggest that, even after restoration of adequate global hemodynamics, cerebral perfusion may still remain markedly impaired as a result of microcirculatory alterations. Even though these data can hardly be confirmed in humans, as a craniotomy would be necessary to investigate the cerebral microcirculation in septic patients, their clinical relevance is important, as cerebral microcirculatory disturbances occur early during the septic process and become even more severe at the moment of shock onset. Further studies are needed to investigate whether cerebral microcirculatory changes are influenced by acute modifications in global hemodynamics during sepsis, such as an increase in MAP or cardiac output using adrenergic agents.

What are the mechanisms associated with these alterations? Microvascular perfusion is regulated by an intricate interplay of many neuroendocrine, paracrine and mechano-sensory pathways [35], adapting to the balance between local oxygen delivery and metabolic needs. Thus, one may imagine that mechanisms implicated in microvascular dysfunction in other organs may also be involved in brain microvascular dysfunction. The recruitment of adherent leukocytes into the microcirculation is a determinant feature of the inflammatory response in different tissues [36,37]. Using intravital microscopy in obese rats, Vachharajani *et al. *showed that cerebral venules presented marked adhesion of platelets and leukocytes to the vascular endothelium already four hours after induced peritonitis, with greatly exaggerated responses in obese mice compared to the lean animals [23]. These endothelial responses were attenuated by selectively blocking adhesion molecules, such as P-selectin, CD18, or intercellular adhesion molecule (ICAM)-1, or by giving dexamethasone [25] or hypertonic solutions [24]. The changes in microvascular density could also be explained by the increased release of nitric oxide (NO) during sepsis [38]. Interestingly, administration of inhibitors of inducible NO synthase (iNOS) prevented cerebral hyperemia [39] but not brain microvascular alterations [22], suggesting that other mechanisms are involved. On the other hand, in cardiogenic and in hemorrhagic shock, the cerebral microcirculation can be preserved even when peripheral microvascular disturbances are present [27,28]. However, the pathophysiology of microcirculatory dysfunction in sepsis may be different and more complex than in these conditions [40,41], and this could explain the cerebral microvascular impairment observed in our study.

What are the consequences of these microvascular alterations? As animals were sedated during the entire experiment and eventually developed fatal sepsis, we were unable to associate microvascular disturbances with clinical neurological abnormalities. Nevertheless, indirect data suggest these alterations may have important consequences on brain cells and function. In endotoxic animals, microcirculatory failure occurred in the early phase of sepsis, and preceded changes in evoked potential responses, indicating that altered perfusion of active neurons is responsible for electrophysiological abnormalities in septic animals [21]. Moreover, cerebral microcirculatory endothelial cells seem to be important structures of the vasoregulative brain system [42], which physiologically maintains a constant CBF during changes in cerebral perfusion pressure [43]. Cerebral autoregulation may be impaired in septic conditions, not only because severe hypotension has been associated with the occurrence of septic encephalopathy [44], but primarily because failure of the CBF to be regulated by changes in MAP or carbon dioxide has already been documented in experimental and human sepsis [7,8,45]. In addition, the septic brain may be more sensitive than other organs to changes in systemic perfusion variables. In septic patients, Berré *et al. *demonstrated that changes in cardiac output induced by dobutamine or prostacyclin were associated with changes in CBF [46,47]. If the microcirculation were also sensitive to or dependent on global hemodynamic changes, then capillary perfusion would have been impaired by a reduction in MAP or improved by an increase in cardiac output. Our results suggest that cerebral autoregulation may be preserved in our septic model, but also that microcirculatory abnormalities in the brain cortex could occur independently from autoregulatory failure, as has already been reported in another experimental study [48]. Finally, microcirculatory alterations may be implicated in the development of cerebral edema. Edema formation has been observed at autopsy in the cortical regions [49] and in the peri-microvascular area, especially in ganglial cells and hippocampal areas, in various experimental models of sepsis [50-52]. However, brain vasogenic edema, caused by a breakdown in the BBB was not observed in the early phase of sepsis in endotoxic rats using magnetic resonance imaging (MRI), despite the presence of microcirculatory disturbances in those animals [53], suggesting that brain edema occurs later and may be a consequence, rather than a cause, of cerebral microcirculatory disturbances.

There are some limitations to this study. First, it was conducted in previously healthy animals under highly controlled conditions, in contrast to the clinical setting in which patients often have underlying illness and co-morbidities, sometimes involving the brain. Second, we did not use antibiotics and vasopressors in order to avoid any influence of these agents on the cerebral microcirculation, although these drugs are currently given in human septic shock. Third, the surgery or the anesthesia provided may have played a role in the observed changes. The absence of microvascular alterations in the sham animals suggests that these procedures were not primarily responsible for these alterations, but they may still have exacerbated the sepsis-induced abnormalities. Moreover, as anesthesia decreases brain metabolism, we may expect an even greater imbalance between microvascular supply and tissue needs in the absence of anesthesia. Fourth, we performed a large bilateral craniectomy that prevented any potential increase in intracranial pressure. However, severe brain swelling has not been reported in several animal and human studies using magnetic resonance imaging (MRI) to study the brain parenchyma during sepsis [53]. Importantly, we avoided local brain injury, minimizing the number of manipulations to four different time points, and taking care to avoid tissue desiccation by local administration of saline solution. Fifth, we visualized only pial vessels and the frontal cortex, and these areas may not be representative of deeper brain structures, including white matter and the brain stem; the velocity of red blood cells in the microvessels was not analyzed. On the other hand, the sheep's brain is also relatively similar to the human brain except for its smaller size and anatomical orientation [54]. Despite the presence of a *rete mirabilis*, the capillary system is similar to the human system and the brain microcirculation images we obtained are quite similar to those shown in the human brain during neurosurgery for subarachnoid hemorrhage [55]. Sixth, we did not measure regional CBF, flow autoregulation or cellular function, and our findings cannot be correlated with cerebral perfusion or metabolism. Moreover, we did not perform any post-mortem histological examination of brain parenchyma. Finally, we could not simultaneously investigate other microcirculations; because of the positioning of the head of the animal (the head was kept in the same position to allow analysis of the same brain region) we could not access the sublingual area. Accordingly, we were unable to evaluate whether these alterations occurred simultaneously with those of other tissues and were of similar severity.

## Conclusions

In this experimental septic shock model, the cerebral microcirculation is impaired, with a significant reduction in perfused small vessels when refractory hypotension and septic shock occur. These alterations may play a role in the pathogenesis of SAE.

## Key messages

• Cerebral microcirculation is progressively impaired during sepsis, with a significant reduction in functional capillary density and proportion of perfused capillaries at shock onset.

• Total vessel density and MFI remain unchanged during the septic process.

• Cerebral microvascular alterations are independent from systemic hemodynamics.

## Abbreviations

ANOVA: analysis of variance; BBB: brain-blood barrier; CBF: cerebral blood flow; CI: cardiac index; FCD: functional capillary density; FIO2: inspired fraction of oxygen; HES: hydroxyethyl starch; ICAM: intercellular adhesion molecule; INOS: inducible nitric oxide synthase; MAP: mean arterial pressure; MFI: mean flow index; MRI: magnetic resonance imaging; NPC: number of perfused capillaries; NO: nitric oxide; OPS: orthogonal polarization spectral; PACO2: arterial pressure of carbon dioxide; PAO2: arterial pressure of oxygen; PAOP: pulmonary artery occlusion pressure; PETCO2: end-tidal carbon dioxide pressure; PSPV: proportion of small perfused vessels; PVD: perfused vessels density; SAE: sepsis-associated encephalopathy; SDF: sidestream dark field; SVR: systemic vascular resistance; TVD: total vessel density.

## Competing interests

The authors declare that they have no competing interests.

## Authors' contributions

FST, FS, JLV and DDB conceived the study protocol. FST, FS, CP, HX, SJ and OD developed the animal model for brain microcirculation analysis. FST, FS, JLV and DDB drafted the present manuscript. All authors read and approved the final manuscript.
